# A multimodal adjoint-state formulation of Quantitative Photoacoustic Tomography with external acoustic sources

**DOI:** 10.1016/j.pacs.2026.100872

**Published:** 2026-08-26

**Authors:** Manne Segerlund, Torbjörn Löfqvist

**Affiliations:** Luleå University of Technology, Department of Computer Science, Electrical- and Space Engineering, Sweden

**Keywords:** Photoacoustic effect, Inverse problem, Image reconstruction, Acoustical heterogenities, Optical heterogenities, Optoacoustic imaging

## Abstract

Quantitative Photoacoustic Tomography (QPAT) aims to recover spatially varying optical and acoustic tissue parameters by utilizing the photoacoustic effect. The associated inverse problem is highly ill-conditioned due to strong coupling between optical absorption, optical scattering, density, and speed of sound. To alleviate the ill-conditioned nature of this problem, an externally generated ultrasonic wavefield is incorporated in addition to photoacoustic sources, resulting in a multimodal formulation of QPAT.

We present a fully coupled, one-step QPAT reconstruction method for the simultaneous recovery of optical absorption, density, and compressibility directly from measurement data, with speed of sound obtained as a derived property. Conventional QPAT and multimodal QPAT arise as special cases within the proposed method. The approach is formulated as a PDE-constrained optimization problem, and the Fréchet derivative of the complete forward model is derived using an optimize-then-discretize adjoint state method. Two-dimensional numerical simulations demonstrate accurate reconstruction of optical absorption, density, and compressibility.

## 1 Introduction

It is well known that Photoacoustic Tomography (PAT) in biological tissue produces high-contrast images due to the combination of strong optical absorption in biomolecules, such as hemoglobin and other chromophores, and a generally low acoustic attenuation. In the subsequent tomographic imaging process, the examined tissue is often considered acoustically homogeneous and isotropic, making the speed of sound in the tissue effectively constant [1]. Frequently used inversion techniques, such as the universal back-projection algorithm, [2] assumes a homogeneous medium with constant speed of sound. This assumption, however, does not strictly hold even in soft tissue [3]. Variations in the speed of sound lead to model discrepancies, which introduces artifacts in the reconstructed tomographic image that are difficult to compensate for or filter out.

In PAT, reconstructing initial pressure for an isotropic heterogeneous medium is possible given prior knowledge of the acoustic properties [4], [5]. However, in the case of non-invasive in vivo imaging, the spatial distribution as well as the acoustic properties of the acoustical heterogeneities are generally not known. Therefore, these parameters must be reconstructed simultaneously with the initial pressure to ensure image integrity [6], [7].

The joint reconstruction of the speed of sound and the initial pressure distribution has been shown to be unstable in the linearized setting [8]. This reflects the inherently ill-conditioned nature of the problem when relying solely on photoacoustic data, and motivates the use of a multimodal method. Here, multimodal refers to a system in which photoacoustic measurements are complemented by independently generated ultrasonic wave fields within the same experimental setup, corresponding to the inclusion of additional acoustic source terms. Such multimodal excitation can be realized either by introducing a light-absorbing, semi-transparent structure that produces an engineered ultrasonic pulse with known characteristics [9], [10], [11], [12], or by employing a conventional ultrasonic transducer activated simultaneously with, or in close temporal proximity to the laser pulse [13].

Considerable work has been devoted to Full Waveform Inversion (FWI) for recovering acoustical heterogeneities [14], [15], [16]. FWI is an inversion technique popularized in geophysics, where it is widely used for seismic imaging. In FWI, the objective is to recover physical parameters directly from the complete recorded wavefield. Ultrasound Full Waveform Inversion (US-FWI), the application of FWI to ultrasound imaging, has been used in both the time domain, based on the acoustic wave equation [17], and the frequency domain, based on the Helmholtz equation [18], [19].

In Quantitative Photoacoustic Tomography (QPAT), the goal is to quantitatively reconstruct the tissue’s optical properties, typically the absorption and scattering coefficients. The optical propagation is most commonly modelled using either the Radiative Transfer Equation (RTE) [20] or its diffusion approximation [21], [22], [23], [24]. The diffusion approximation is known to lose accuracy in weakly scattering or near-boundary regimes [20], namely in regions closest to the source and therefore of greatest relevance to QPAT. However, the initial pressure depends only on the total deposited optical energy, so discrepancies between the RTE and its diffusion approximation are observable only through their effect on the resulting initial pressure field.

QPAT can be divided into two main approaches: one-step [24] and two-step [20], [21], [23] methods. In a two-step approach, the initial pressure is first reconstructed using PAT, after which the optical inversion is solved such that the modelled initial pressure matches the reconstructed initial pressure. In a one-step approach, the optical parameters are reconstructed directly from the measurement data without the intermediate reconstruction of the initial pressure. From an inversion perspective, one-step methods offer several advantages, albeit at the additional computational cost of solving a coupled optical and acoustic inverse problem. When reconstructing the initial pressure $p_{0}\in L^{2}\left(\Omega \right)$ independently, the reconstruction does not account for the restrictions imposed by the RTE on admissible initial pressures. Consequently, the intermediate space of reconstructed initial pressures is larger than the set of physically realizable photoacoustic sources permitted by the coupled optical model. Furthermore, when using multiple illuminations from different directions, which is common in QPAT, each illumination introduces an additional initial pressure reconstruction despite the optical absorption and scattering parameters remaining unchanged across illuminations. Consequently, the number of intermediate unknowns grows with the number of illuminations, even though the underlying optical parameters remain fixed.

In this work, we extend the framework of QPAT to quantitatively recover acoustic parameters, namely density and compressibility, with the speed of sound treated as a derived property. Inspired by both geophysical FWI and one-step QPAT, the proposed formulation yields a fully coupled acoustic and optical inversion framework in which conventional QPAT, US-FWI, and multimodal excitation arise naturally as different source configurations of the same underlying formulation. The absorption and reduced scattering coefficients are reconstructed using a one-step inversion approach. The proposed method is derived using an operator-level formulation of the adjoint-state method following an Optimize-Then-Discretize (OTD) methodology, using the diffusion approximation for the optical model and the linear acoustic wave equation without attenuation.

The PDEs are solved using the Finite Element Method (FEM). The photon diffusion equation is solved using standard FEM discretization techniques. The acoustic wave equation is solved using a hybridized Discontinous Galerkin Finite Element Method (DG-FEM) formulation [25], [26]. The method is designed to scale efficiently for large three-dimensional problems, leveraging a fully distributed formulation that avoids global operations and is well suited for high-performance computing environments.

A numerical test problem is included to demonstrate the feasibility of the method in a simple setting, while further work is required to fully explore its applicability and performance in realistic three-dimensional scenarios. To demonstrate the flexibility of the formulation, four different setups are considered. These correspond to different inversion methods. In the present test case, the Grüneisen parameter and the diffusion coefficient are held constant, though the method is general and can accommodate inversion of these parameters in future studies.

The remainder of this paper is organized as follows. Section 2 introduces the mathematical models for the complete photoacoustic process. Section 3 derives the corresponding adjoint equations and gradient expressions. Section 4 describes the regularization and optimization strategy. Section 6 demonstrates the proposed framework using the considered test problems. Finally, the results are discussed in Section 7, and conclusions are drawn in Section 8.

## 2 Modelling the photoacoustic effect

Modelling the photoacoustic effect and the connected forward problems, involves modelling the physical process of light scattering and absorption, the thermoelastic conversion of absorbed light energy to pressure and the propagation of acoustic waves in tissue.

Given that the laser pulse length is typically in the nanosecond range, it is appropriate to represent the laser light illumination as being in steady state for modelling the optical scattering and absorption for each pulse separately.

Photon transport in biological tissue can be modelled using RTE, which is equivalent to the Monte Carlo method [27]. If assuming that photon scattering is much more frequent than photon absorption, which means that each photon will be scattered multiple times before it is absorbed, the diffusion approximation of the RTE can be used [27],

(1) $$\left\{\begin{array}{cc} \mu \phi -\nabla \cdot \left(D\nabla \phi \right)=0\; & \text{in}\;\Omega \\ \phi +2D\nabla \phi \cdot n=j\left(x\right)\; & \text{on}\;\partial \Omega , \end{array}\right)$$

where ϕ=ϕ(x) is the light intensity while μ=μ(x) and D=D(x) denote the spatially varying optical absorption and diffusion coefficients μ the optical absorption coefficient and D the diffusion coefficient from the diffusion approximation of RTE. Ω represents the illumination area and therefore the QPAT domain. The boundary function j(x) represents the light entering the domain through the boundary due to the laser illumination. While some QPAT studies in the diffusion approximation impose Dirichlet boundary conditions for the photon fluence [21], [23], we employ a Robin boundary condition [28]. This provides a physically consistent boundary model that accounts for incoming and outgoing diffusive flux, while avoiding the need to know the true photon fluence at the boundary, which depends on unknown internal parameters. If there is no illumination and the measurement represent a pure ultrasound measurement, this can be achieved by assigning j=0 in (1), which will result in ϕ=0.

If the laser pulse is temporally much shorter than r/c, where r is the spatial resolution and c is a representative speed of sound for the tissue, then stress confinement is an appropriate approximation for the acoustic wave equation. For a spatial resolution of the order of 100 μm the stress confinement time-scale is approximately 67 ns. A typical laser pulse duration is less than 10 ns [29] which means that stress confinement is an appropriate assumption. Assuming stress confinement, the initial pressure can then be expressed as

(2) $$p_{0}=\Gamma \mu \phi ,$$

where Γ=Γ(x) is the Grüneisen parameter describing the conversion from absorbed laser energy to pressure.

The elastic wave propagation is modelled using the linear acoustic wave equation, expressed as a system of linear equations in pressure p=p(x,t) and particle velocity v=v(x,t),

(3) $$\left\{\begin{array}{c} \frac{1}{\rho c^{2}}\frac{\partial p}{\partial t}+\nabla \cdot v=f\;\\ \rho \frac{\partial v}{\partial t}+\nabla p=0\;\\ p\left(x,0\right)=\Gamma \mu \phi \;\\ v\left(x,0\right)=0\; \end{array}\right)$$

The QPAT domain is embedded into an infinite continuous domain such that $p\in C^{\infty }\left(R^{d}\right)$ and $v\in {\left(C^{\infty }\left(R^{d}\right)\right)}^{d}$. The infinite domain is truncated numerically using a Perfectly Matched Layer (PML) [30]. Here, both the speed of sound and the density are spatially varying, with c=c(x) assumed non-dispersive and ρ=ρ(x). The acoustic source injected into the domain is represented by the source term f. In a QPAT setup, no external acoustic source is present and this can be represented by setting f=0 in (3).

An observer operator P is applied to the solution of the acoustic wave equation. It is assumed to be bounded, linear, and time-invariant, and therefore admits a well-defined Hilbert adjoint $P^{*}$. The operator P maps the acoustic state u=(p,v), obtained from the forward model, to the measurement domain representing the action of the acquisition process. It may act on pressure, particle velocity, or both; no further assumptions on its structure are required.

Measurements can be repeated for multiple pulses with different illumination or acoustic source directions, and so on, to increase the information that can be extracted from the data.

## 3 Fréchet derivative expressions

The imaging problem is formulated as an optimization problem, in which the model parameters are adjusted to minimize a suitable objective function. For brevity, the Fréchet derivative will be referred to simply as the derivative in the following text.

Following standard practice in full-waveform inversion and QPAT using the adjoint state method, the objective function is defined as the least-squares difference between the observed data (d) and the modelled data (P(u)), where u denotes the acoustic state obtained from the forward model. Formally, the objective function can then be written as

(4) $$J=\frac{1}{2}\|P\left(u\right)-d{\|}^{2}.$$

The optimization problem is then formulated as

(5) $$\underset{m}{\min}\;J\left(\mu ,D,\Gamma ,c,\rho \right),$$

where m=(μ,D,Γ,c,ρ), or any subset of these parameters, subject to the positivity constraint

$$m_{i}\left(x\right)>0,\;\forall \;x\in \Omega ,$$

for each estimated parameter $m_{i}$.

To simplify the derivative with respect to the acoustic parameters, we introduce auxiliary variables α and β to (3), chosen such that the wave operator depends linearly on them. This is a mathematical reparameterization to simplifying the derivative, rather than a physically motivated change of variables. For the acoustic wave equations, this choice corresponds to $\alpha =1/\rho c^{2}$ and β=ρ so that the operator becomes linear in (α,β). In this case, β coincides with the original density and α corresponds to compressibility. The speed of sound c is therefore not inverted directly, but recovered implicitly through the relation $c={\left(\alpha \beta \right)}^{-1/2}$. This formulation allows derivatives for any given parameterization of the wave equation to be obtained by applying the chain rule to the derivatives with respect to α and β. With this parameterization, the derivative of the operator reduces to simple time-derivative terms, and the wave equation (3) takes the form

(6) $$\left\{\begin{array}{c} \alpha \frac{\partial p}{\partial t}+\nabla \cdot v=f\;\\ \beta \frac{\partial v}{\partial t}+\nabla p=0\;\\ p\left(r,0\right)=\Gamma \mu \phi \;\\ v\left(r,0\right)=0.\; \end{array}\right)$$

A complete derivation of the adjoint state equations and derivative, including operator-level product rules and chain-rule linearizations, is provided in Appendix A. Here, we present only the resulting equations.

The acoustic adjoint equation with time reversed fields ${\overset{ˆ}{p}}^{*}$ and ${\overset{ˆ}{v}}^{*}$ is expressed as

(7) $$\left\{\begin{array}{c} \alpha \frac{\partial {\overset{ˆ}{p}}^{*}}{\partial t^{\prime }}+\nabla \cdot {\overset{ˆ}{v}}^{*}=-q_{p}\left(T_{f}+t_{0}-t^{\prime }\right)\;\\ \beta \frac{\partial {\overset{ˆ}{v}}^{*}}{\partial t^{\prime }}+\nabla {\overset{ˆ}{p}}^{*}=q_{v}\left(T_{f}+t_{0}-t^{\prime }\right)\;\\ {\overset{ˆ}{p}}^{*}\left(x,0\right)=0\;\\ {\overset{ˆ}{v}}^{*}\left(x,0\right)=0\; \end{array}\right)$$

with

$$\left(q_{p},q_{v}\right)=P^{*}\left(P\left(u\right)-d\right).$$

Which is the acoustic wave equation running backwards in time from $T_{f}$ to $t_{0}$ with the residual $P^{*}\left(P\left(u\right)-d\right)$ injected as a source term. The adjoint solutions are then defined as,

$$\left(\begin{array}{c} p^{*}\left(x,t\right)\\ v^{*}\left(x,t\right) \end{array}\right)=\left(\begin{array}{c} {\overset{ˆ}{p}}^{*}\left(x,T_{f}+t_{0}-t\right)\\ -{\overset{ˆ}{v}}^{*}\left(x,T_{f}+t_{0}-t\right) \end{array}\right).$$

The photon diffusion adjoint equation is the elliptical equation with Robin boundary terms defined as,

(8) $$\left\{\begin{array}{cc} \mu \phi^{*}-\nabla \cdot \left(D\nabla \phi^{*}\right)=\Gamma \mu p^{*}\left(x,t_{0}\right)\; & \text{in}\;\Omega \\ \phi^{*}+2D\nabla \phi^{*}\cdot n=0\; & \text{on}\;\partial \Omega . \end{array}\right)$$

The adjoint photon diffusion equation is identical to the photon diffusion equation to the difference of a homogeneous Robin boundary condition and a source term. The same is true for the acoustic equation. Consequently, the same numerical solvers can be used for both forward and adjoint problems.

Applying (A.1) with the solutions to the adjoint state equations, the derivatives of the objective function are expressed as

(9) $$\begin{array}{cc} D_{\mu }J\;\delta \mu & =\int_{\Omega }\delta \mu \left(\phi \phi^{*}-\Gamma \phi p_{0}^{*}\right)dV \end{array}$$

(10) $$\begin{array}{cc} D_{\Gamma }J\;\delta \Gamma & =-\int_{\Omega }\delta \Gamma \mu \phi \;p_{0}^{*}dV \end{array}$$

(11) $$\begin{array}{cc} D_{D}J\;\delta D & =-\int_{\Omega }\nabla \cdot \left(\delta D\;\nabla \phi \right)\;\phi^{*}dV\\ & =\int_{\Omega }\delta D\nabla \phi \cdot \nabla \phi^{*}dV \end{array}$$

(12) $$\begin{array}{cc} D_{\alpha }J\;\delta \alpha & =\int_{\Omega }\int_{t_{0}}^{T_{f}}\delta \alpha \frac{\partial }{\partial t}pp^{*}dtdV \end{array}$$

(13) $$\begin{array}{cc} D_{\beta }J\;\delta \beta & =\int_{\Omega }\int_{t_{0}}^{T_{f}}\delta \beta \frac{\partial }{\partial t}v\cdot v^{*}dtdV. \end{array}$$

Here, δμ, etc., denote perturbations to the model parameters. For multiple illumination pulses or sources, the total derivative of the objective function with respect to the model parameters is obtained by summing the contributions from each source individually,

$$D_{m}J=\overset{N_{s}}{\underset{i=1}{\sum }}D_{m}J_{i}.$$

Each source is treated independently in the forward and adjoint simulations, and the linearity of the derivative allows these contributions to be accumulated.

The derivative derived here is a functional, an element of the dual of the parameter space, and therefore cannot be added directly to the current state in a gradient-based optimization method, doing so would be a categorical error. To update the model, the derivative must be mapped back to the primal space. This is possible if the model parameters are defined in a Hilbert space, using the Riesz isomorphism. Accordingly, the gradient (∇J) is defined as the unique element of the primal space that the Fréchet derivative maps to via the inverse Riesz map such that [31],

$$D_{m}J\delta m=\langle \nabla J,\;\delta m\rangle ,\;\forall \delta m\in A$$

where δm denotes an arbitrary admissible perturbation of the parameter field m, and A is the Hilbert space of admissible parameter fields.

We define the parameter inner product space A for the inversion parameters with the inner product

$${\langle u,\;v\rangle }_{A}=\underset{i}{\sum }\int_{\Omega }w_{i}\left(x\right)\;u_{i}v_{i}+{ɛ}_{i}{\left(x\right)}^{2}\;\nabla u_{i}\cdot \nabla v_{i}\;dV,$$

with the induced norm $\|u{\|}_{A}=\sqrt{{\langle u,\;u\rangle }_{A}}$. Here, $w_{i}\left(x\right)>0$ is a strictly positive, spatially varying weighting function, and ${ɛ}_{i}\left(x\right)\geq 0$ is a spatially varying correlation length controlling smoothing.

In this formulation, the weighting and smoothing act as tuneable preconditioners for the model update: locally high weights make large updates costly in terms of the norm, suppressing changes in that region, while small weights increase the magnitude of updates. The smoothing parameter ${ɛ}_{i}$: larger values produce stronger smoothing, while smaller values preserve local variations.

In practice, once the parameter inner product space is discretized using the chosen basis functions, the inverse Riesz map reduces to solving a linear system. Denoted as A, the matrix representation of the inner product bilinear form, the discrete gradient g corresponding to the derivative DJ is obtained by solving

Ag=DJ.

where DJ is the derivative evaluated on each basis function.

Without mapping the derivative to the primal space via the Riesz isomorphism, the magnitude of the update would primarily reflect the size of the discretization cells rather than the local sensitivity of the measurements. In practice, the inverse Riesz map ensures that updates are properly scaled according to the chosen inner product, incorporating local sensitivity and smoothing.

The use of an OTD methodology is consistent with the operator-level formulation adopted here. Since the gradient is defined through the continuous inversion-space geometry and corresponding Riesz map, different discretizations approximate the same underlying gradient structure, with discrepancies arising only from the discretization error itself. This is particularly advantageous for the DG-FEM formulation used in this work, where the flux variables introduce globally coupled discrete quantities. A Discretize-Then-Optimize (DTO) formulation would instead depend explicitly on the chosen discretization and time integration scheme, increasing complexity and reducing generality.

## 4 Regularization and stabilization

The following strategies are employed to stabilize the inversion and improve convergence. These techniques are purely numerical: they do not introduce additional physical assumptions, but rather guide the optimization to produce physically consistent solutions.

To stabilize the inversion, the objective function is augmented with a regularization term:

$$J^{\prime }\left(m\right)=J\left(m\right)+R\left(m\right),$$

$$D_{m}J^{\prime }\left(m\right)=D_{m}J\left(m\right)+D_{m}R\left(m\right).$$

Regularization can reduce the ill-conditioned nature of the problem and improve convergence by introducing prior knowledge about the solution, such as smoothness or piecewise constancy.

For heterogeneous tissue, we assume that the domain is composed of regions with nearly constant material properties, separated by sharp interfaces. For such an assumption, a Total Variation (TV) regularization is appropriate [32]. Here we use the isotropic TV defined as,

$$R_{TV}\left(m\right)=\underset{m_{i}}{\sum }\lambda_{i}\int_{\Omega }\|\nabla m_{i}{\|}_{2}dV,$$

where $\lambda_{i}$ is the weighting term for parameter i. The TV regularization can be smoothed out by using a small parameter ɛ>0:

$$R_{TV}\left(m\right)=\underset{m_{i}}{\sum }\lambda_{i}\int_{\Omega }\sqrt{\|\nabla m_{i}{\|}_{2}^{2}+{ɛ}^{2}}\;dV.$$

which yields the derivative in the direction δm

$$D_{m}R_{TV}\left(m\right)\;\delta m=\underset{m_{i}}{\sum }\lambda_{i}\int_{\Omega }\frac{\nabla m_{i}\cdot \nabla \left(\delta m_{i}\right)}{\sqrt{\|\nabla m_{i}{\|}_{2}^{2}+{ɛ}^{2}}}dV.$$

In the DG-FEM discretization used for the model parameters, the fields $m_{i}$ are discontinuous across element interfaces. To account for this, the TV regularization is extended to include both intra-element gradients and inter-element jumps:

$$R_{TV}\left(m\right)=\underset{m_{i}}{\sum }\lambda_{i}\left[\underset{K\in T}{\sum }\int_{K}\sqrt{\|\nabla m_{i}{\|}_{2}^{2}+{ɛ}^{2}}\;dV+\right)\left(\underset{\partial K\in T^{i}}{\sum }\int_{\partial K}\sqrt{{\left[m_{i}\right]}^{2}+{\left(h_{K}ɛ\right)}^{2}}\;dS\right].$$

Here, T denotes the set of elements, $T^{i}$ the set of interior faces, [⋅] the jump across a face, and $h_{K}$ a characteristic length scale associated with the face ∂K. The derivative becomes

$$D_{m}R_{TV}\left(m\right)\;\delta m=\underset{m_{i}}{\sum }\lambda_{i}\left[\underset{K\in T}{\sum }\int_{K}\frac{\nabla m_{i}\cdot \nabla \left(\delta m_{i}\right)}{\sqrt{\|\nabla m_{i}{\|}_{2}^{2}+{ɛ}^{2}}}dV+\right)\left(\underset{\partial K\in T^{i}}{\sum }\int_{\partial K}\frac{\left[m_{i}\right]\left[\delta m_{i}\right]}{\sqrt{{\left[m_{i}\right]}^{2}+{\left(h_{K}ɛ\right)}^{2}}}\;dS\right].$$

Full waveform inversion is a non-convex optimization problem, meaning that the objective function may have multiple local minima. The non-convex nature is due, in part, to a phenomena called cycle skipping. If a model predicts the arrival of a synthetic wave 2π out of phase relative to the measured arrival, then, to correct this phase error, the objective function will increase when the arrival time is shifted by a small amount. Hence, the process of full waveform inversion is a non-convex optimization problem [33]. Consequently, gradient-based optimization method may get trapped in a local minimum before finding the global minimum. In frequency domain inversion techniques, it is common to invert low frequencies first, which has been shown to lead to an improvement in inversion results [18]. Another approach is a multiscale method, as in [34]. In such methods, the mesh is refined from a coarse mesh to a fine mesh, gradually increasing the resolution of the image. Starting with basis functions with large support allows the inversion to capture the large-scale features of the solution, while progressively introducing basis functions with smaller support provides finer-scale details. Multiscale methods also have the advantage of using a coarser meshes initially, leading to faster simulations early in the inversion process, and fewer parameters tends to lead to better conditioned inversion. A strategy analogous to inverting low frequencies first in the frequency domain can be applied in time-domain methods by filtering the measured residual with a low-pass filter. This modifies the objective function to

$$J_{f}=\frac{1}{2}\|H_{f}\left(P\left(u\right)-d\right){\|}^{2}$$

where $H_{f}$ is a linear time-invariant low-pass filter applied independently to the residual of each sensor, emphasizes large-scale (low-frequency) components of the mismatch. This guides the optimization to first fit the broad structures of the model, reducing the risk of being trapped in local minima associated with high-frequency features, before progressively incorporating finer-scale details. Since $H_{f}$ is linear and time-invariant, its adjoint is given by cross correlation with the same impulse response. Because the filter is applied in post-processing, it can be chosen to be self-adjoint, so that $H_{f}^{*}=H_{f}$, further simplifying the computation of the adjoint source:

$$\left(q_{p},q_{v}\right)=P^{*}\left(H_{f}^{2}\left(P\left(u\right)-d\right)\right).$$

Low pass filter and multiscale methods combines well with an adaptive refinement approach to FEM. Low pass filtering is used to smooth out the objective function, while the adaptive refinement approach refines the mesh at the locations with high sensitivity to use the computational resources in the most efficient way possible.

The optimization problem is solved using a Limited-memory Broyden–Fletcher-Goldfab-Shanno (L-BFGS) [35] method with backtracking Armijo line search [36]. The gradient supplied to the optimizer is obtained via the inverse Riesz map corresponding to the chosen parameter inner product. Secant pairs are evaluated consistently with this inner product to preserve the induced Hilbert-space geometry. Simple bound constraints are enforced by projection after each update.

## 5 Implementation

The QPAT method is implemented using the general-purpose finite element library deal.II [37], [38]. The acoustic wave equation is solved using a hybridized discontinuous Galerkin formulation [25] with explicit Runge–Kutta time integration. The photon diffusion equation is solved using the standard practice of FEM discretization. Lagrange interpolation polynomials are used as basis functions for the finite element spaces. The initial pressure distribution is obtained by evaluating Γμϕ at the support points of the Lagrange basis functions, thereby defining its Lagrange interpolation. Revolve [39] is used for checkpointing of the forward solution.

The derivative integrals for each parameter basis are evaluated using standard quadrature, allowing arbitrary polynomial degree for the parameter fields. This enables the use of polynomial parameter fields (e.g., linear basis functions or higher order polynomials) rather than piecewise constant representations, while maintaining consistency of the Fréchet derivative and inverse Riesz map. In this setting, the inverse Riesz map is essential, as the parameter basis is not orthogonal and the gradient must be mapped consistently to the primal space.

The implementation is fully distributed and supports parallel execution on distributed-memory architectures using the Trilinos wrapper [40] for the photon diffusion equation and the deal.II matrix-free framework for the acoustic wave equation.

## 6 Numerical test problem

### 6.1 Test problem design

The test problem is a 2D cylindric domain with a circular array of ultrasound sensors surrounding the entire domain to eliminate the limited view problem [41].

**Fig. 1:**
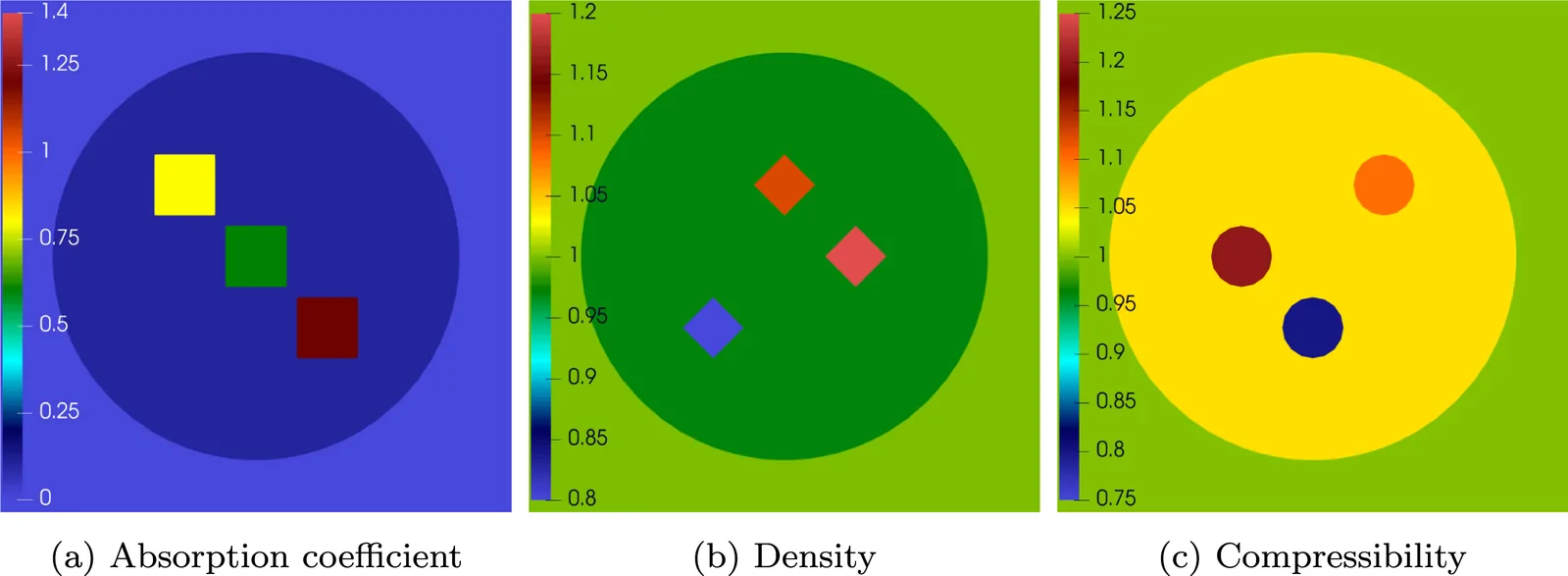
Test problem. The anomalies in each parameter field are placed at different locations.

**Fig. 2:**
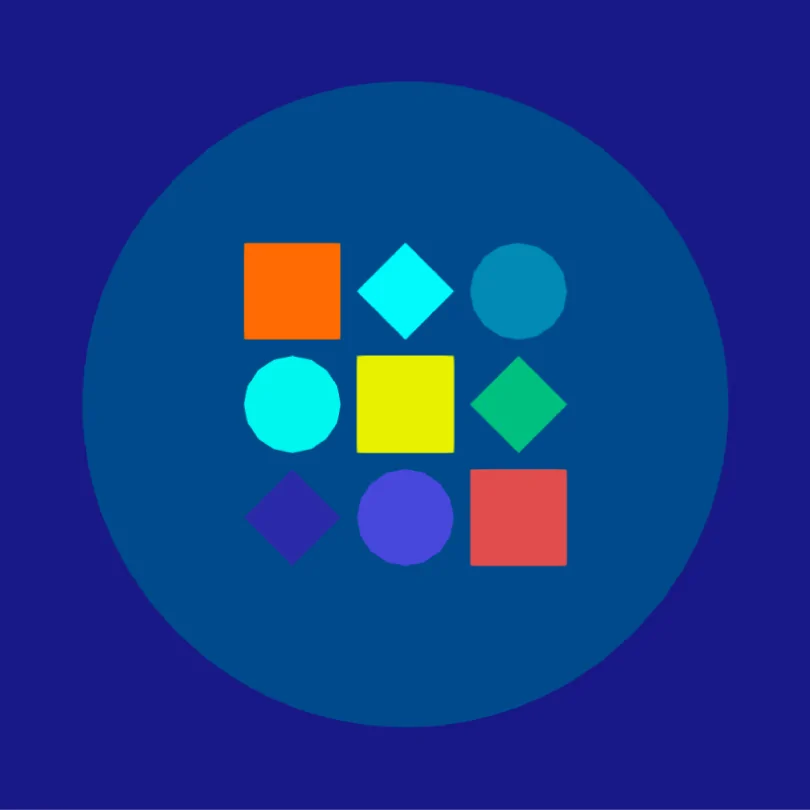
Composite visualization (sum of absorption coefficient, density, and compressibility). The values are not physically meaningful and are used only to illustrate the spatial separation of anomalies across parameters. This separation highlights that any overlapping structures observed in the reconstructions arise from cross-talk rather than true features.

The test problem is designed such that the regions in which μ,α, and β differ from the background $\mu_{0},\alpha_{0}$, and $\beta_{0}$ are located in separate areas of the domain. This configuration highlights potential cross-talk between the parameters. As a result, the inversion of μ,α, and β is more challenging than might be expected from a geometry of this simplicity. An illustration of the test problem is provided in Fig. 1. A composite visualization is provided in Fig. 2, where the non overlapping anomalies are clearly visualized.

The Grüneisen parameter is assumed to be constant and known. This assumption is made due to the sensitivities of the absorption coefficient and the Grüneisen parameter are very similar; their effects can only be distinguished through the effects of variations in the absorption coefficient’s influence on the light intensity in different regions of the domain. Accounting for both parameters simultaneously would therefore make an already ill-conditioned problem even more ill-conditioned.

The observer operator, its adjoint, and the data space inner product are defined in Appendix B. They map the continuous acoustic field to the discrete sensor measurements $\left\{p_{ij}\right\}$ and vice versa. Their discrete realization follows directly by applying the standard spatial and temporal quadrature rules used in the finite element discretization. The formulation itself does not restrict the choice of P, and alternative models, such as band-limited sampling or temporally integrated measurements, can be incorporated without modification of the adjoint derivation.

All parameters are expressed in dimensionless form. The laser illumination is modelled using a Gaussian profile with a Full Width Half Maximum (FWHM) of 0.2 length units. A band-limited acoustic pulse (central frequency 10 time^−1^) is used for excitation, chosen to balance sensitivity and mitigate cycle skipping. The background optical parameters are selected to satisfy the diffusion approximation. A total of 20 simulations are performed. In each simulation, the laser illumination and the acoustic source centred at the same angular position on the boundary of the QPAT domain. This common position is rotated uniformly around the boundary in increments of 360°/20, resulting in 20 distinct illumination–acoustic source configurations. A total of 100 sensors are uniformly distributed around the domain.

**Fig. 3:**
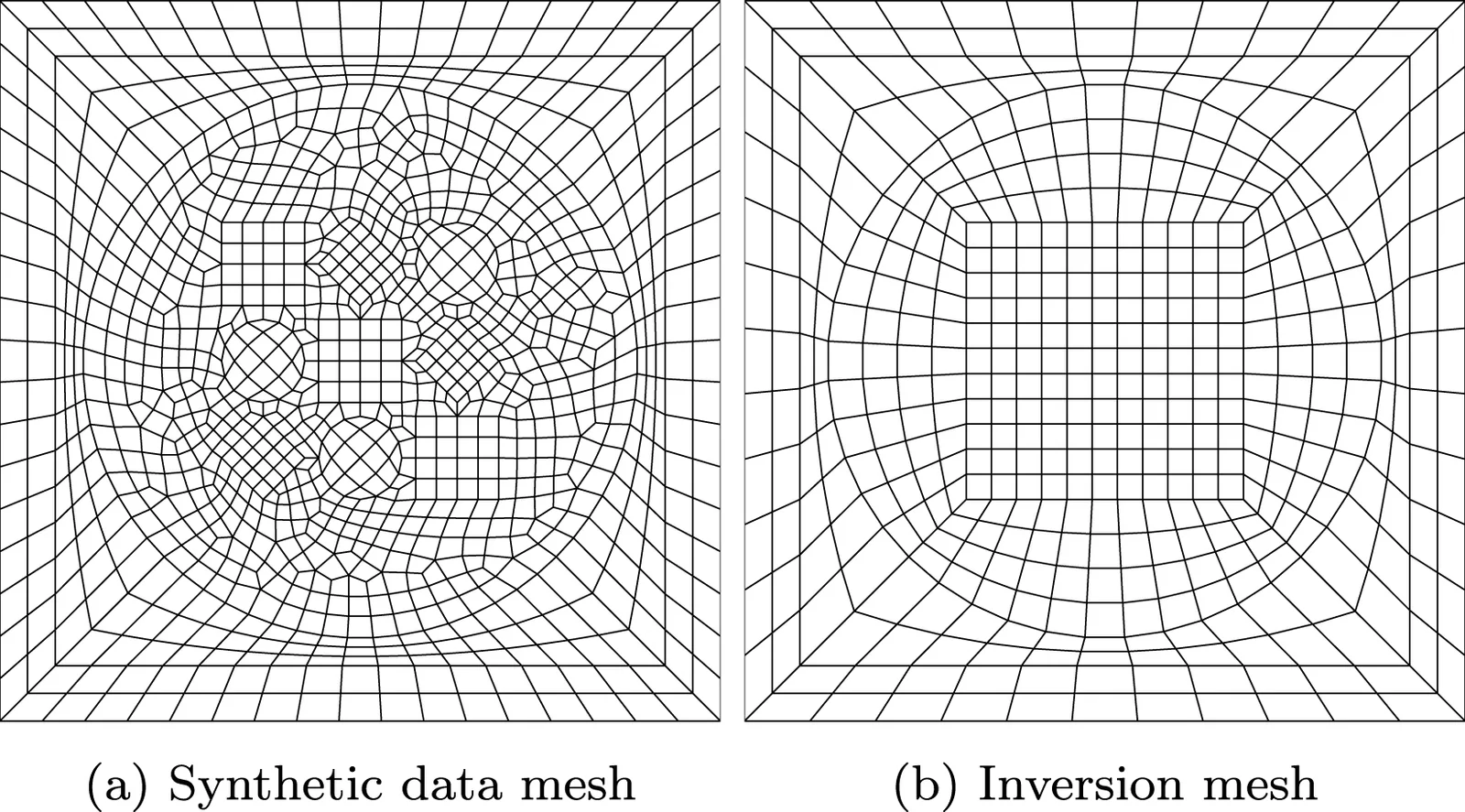
Computational meshes used in the numerical test problem. The inversion mesh does not align with the data mesh, illustrating mesh independence and avoiding inverse crime effects. The circular region indicates the QPAT domain.

To avoid the inverse crime [42], different meshes are used for data generation and inversion as can be seen in Fig. 3, with adaptive refinement. White Gaussian noise (1% of the pressure amplitude) is added to the data.

Interpolation polynomials of degree 2 are used for the state variables and linear polynomials for the model parameters. A constant weight of 1 is used, and a constant smoothing parameters based on the median length scale h of the cells in the discretization. This provides modest smoothing without significantly altering local variations.

The low pass filter is defined in Appendix B. When the model has converged for a given cutoff frequency, or if the gradient fails to produce a descent direction, the cutoff frequency is increased and the optimization is restarted from the current parameter estimate. At this stage, the mesh is also refined using a Kelly-type error indicator [43].

The regularization weight is chosen such that the data misfit gradient and the regularization gradient have comparable norms at each restart,

$$\lambda_{i}=\frac{\|\nabla J_{i}\left(m_{k}\right){\|}_{A}}{\|\nabla R_{i}\left(m_{k}\right){\|}_{A}},$$

where $m_{k}$ denotes the current parameter state at the restart.

The test problem is evaluated for four configurations: homogeneous QPAT, QPAT, US-FWI, and multimodal QPAT. In homogeneous QPAT, only the absorption coefficient is treated as an active inversion parameter, and only optical illumination is used as the source. The acoustic parameters are therefore fixed at their initial values of 1 for both compressibility and density. In QPAT, absorption, compressibility, and density are all treated as active inversion parameters, but only optical illumination is used. In US-FWI, absorption, compressibility, and density are likewise active inversion parameters, but only acoustic sources are used. Finally, multimodal QPAT considers all three parameters as active inversion parameters while employing both optical illumination and acoustic sources. These configurations are summarized in Table 1. To ensure a fair comparison, all cases are optimized using an identical optimization configuration, including the same regularization parameters, convergence criteria, and solver settings. Only the source configuration is varied to reflect the different inversion methods.

**Table 1:** Summary of the inversion configurations used in the test problem.

Method	Illumination	External wavefield	Acoustic fitting
Homogeneous QPAT	✓		
QPAT	✓		✓
US-FWI		✓	✓
Multimodal QPAT	✓	✓	✓

### 6.2 Results

The results obtained from the different inversion methods are analysed separately for each parameter. The purpose of the following comparisons is not to identify a single superior inversion method, but rather to demonstrate that the proposed mathematical framework encompasses multiple inverse problems through different source configurations and sets of active parameters. The methods are therefore analysed primarily to illustrate the flexibility and generality of the formulation, rather than to establish a quantitative ranking between them. Consequently, quantitative reconstruction accuracy metrics are not reported, as establishing a quantitative ranking between the methods is not the objective of this study. Instead, the comparisons focus on characteristics such as parameter recoverability, cross-talk between parameters, artifact suppression, and reconstruction of anomaly location and contrast, which are more informative for assessing the influence of the different inversion methods.

**Fig. 4:**
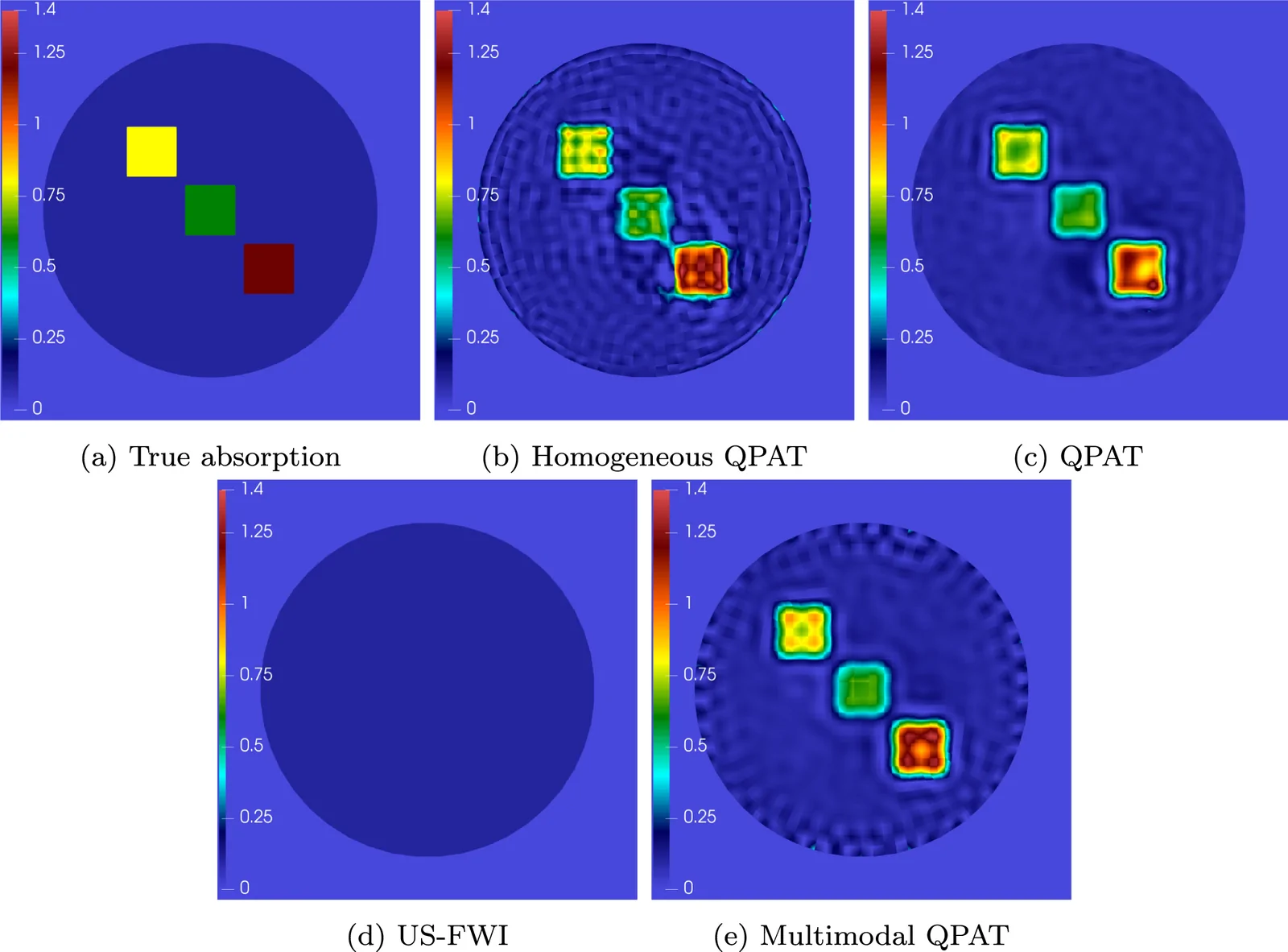
Absorption coefficient inversion results. The multimodal QPAT result shows more symmetric anomalies than the QPAT result. Because no illumination is present in US-FWI, the sensitivity to the absorption coefficient is zero and thus the search becomes identically zero.

We begin with the absorption coefficient, shown in Fig. 4. The reconstruction obtained under the assumption of homogeneous acoustic properties is noticeably worse than the remaining approaches. In particular, the central (0.6) square appears rectangular and is shifted slightly toward the lower right. Both the QPAT and the multimodal QPAT show good agreement with the true absorption. Both reconstructions also exhibit signs of the Gibbs phenomenon, visible as oscillations near the boundaries. The structures recovered by the multimodal QPAT appear more square-like, which may be attributed to the improved reconstruction of the acoustic parameters. This, in turn, enables more accurate localization of the photoacoustic sources. The US-FWI reconstruction remained equal to the initial guess throughout the inversion. Since no photoacoustic source is present in US-FWI, the objective functional has no sensitivity to the absorption coefficient, and the corresponding search direction therefore remains identically zero.

**Fig. 5:**
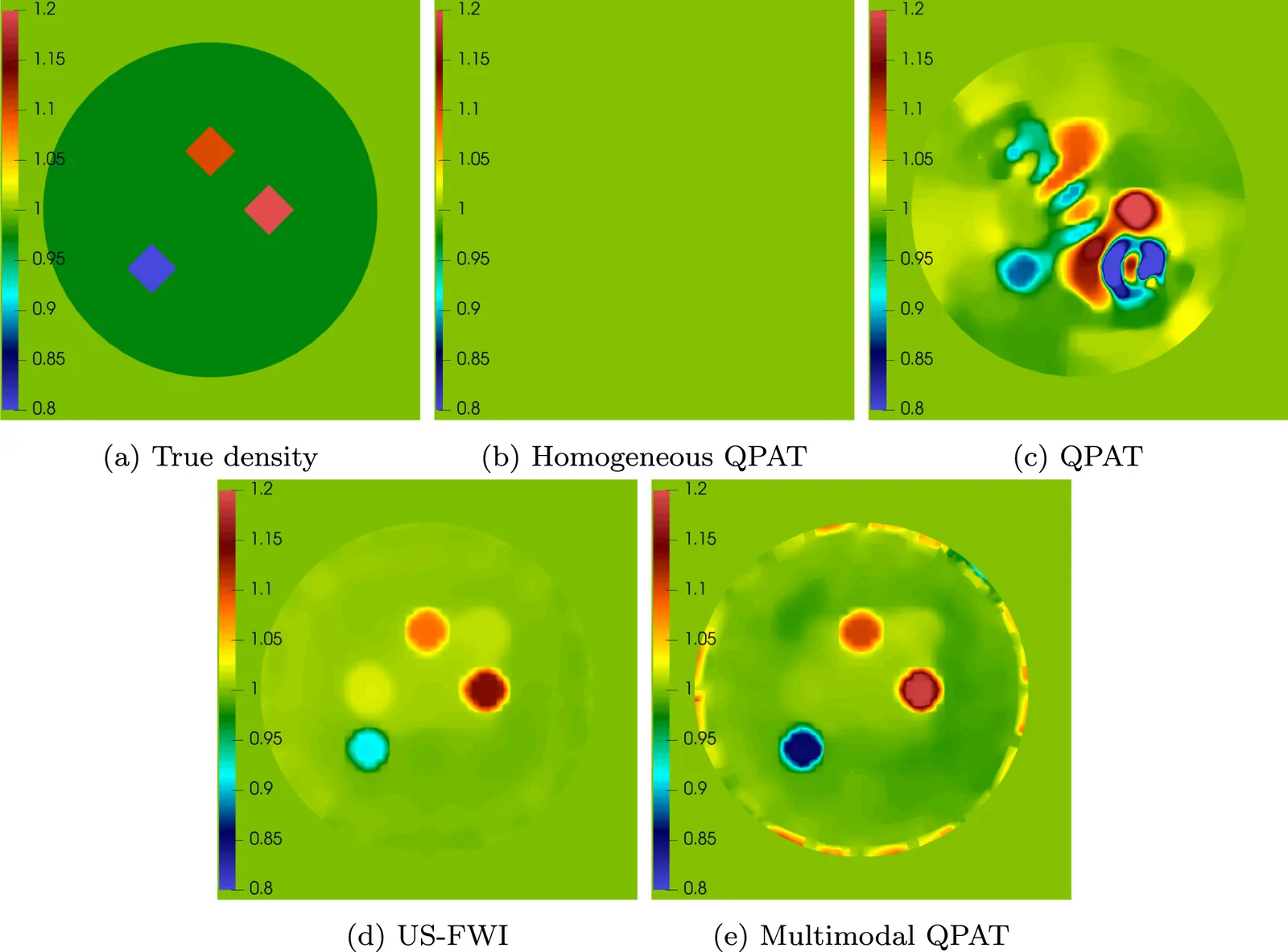
Density inversion results. The QPAT method fails to reconstruct the density due to the unstable nature of the inverse operator. The US-FWI method successfully recovers the anomalies, with slight cross-talk with the compressibility. The multimodal QPAT also successfully reconstructs the density, with reduced cross-talk and improved recovery of the background density variation. The reduced cross-talk is evidenced by the suppression of the circular yellow feature present in the US-FWI result, which is not observed in the multimodal QPAT result.

The inversion results for the density are shown in Fig. 5. The QPAT fails to reconstruct the density, which can be attributed to the instability of the inverse operator when relying solely on the initial pressure. Significant cross-talk with the absorption coefficient is evident from artifacts at the locations of the absorption anomalies. The US-FWI method reconstructs the density anomalies, although the background shows a slight bias toward the initial guess. A faint circular artifact is also present, corresponding to cross-talk with the compressibility (1.2) anomaly, although its amplitude is low. Finally, the multimodal QPAT achieves a good reconstruction of the density anomalies with reduced cross-talk with the compressibility, as evidenced by the absence of the circular artifact observed in the US-FWI result. The background is also closer to the true density, although a slight bias toward the initial guess remains.

**Fig. 6:**
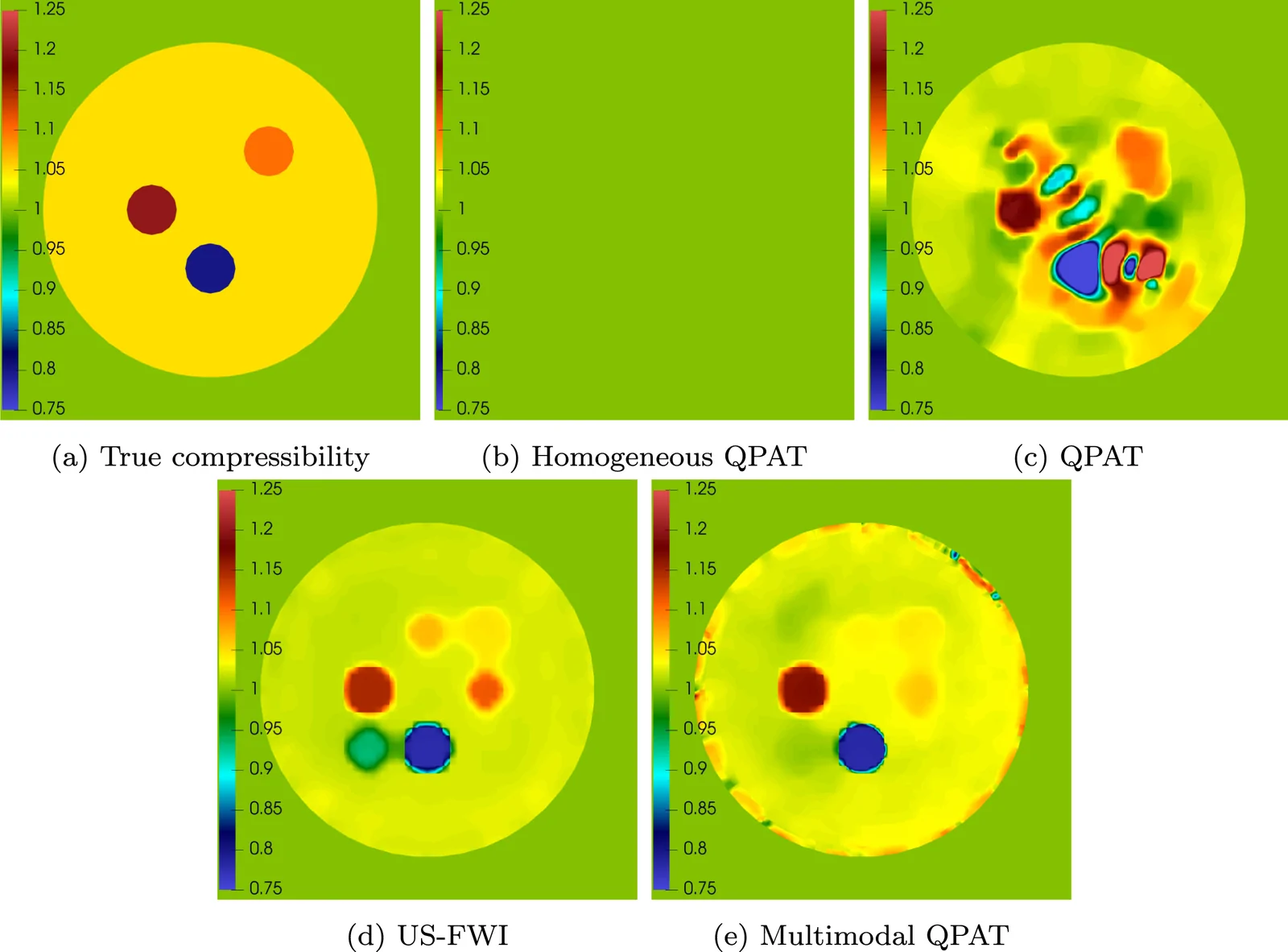
Compressibility inversion results. The QPAT method fails to reconstruct the compressibility accurately due to the unstable nature of the inverse operator. The US-FWI method recovers the anomalies, with slight cross-talk with the density. The multimodal QPAT also successfully reconstructs the compressibility, with reduced cross-talk as evidenced by the suppression of the spurious green diamond.

The inversion results for the compressibility follow a similar trend to the density, as seen in Fig. 6. QPAT fails to reconstruct the parameter, consistent with the instability observed when relying solely on the initial pressure. The US-FWI method recovers the main anomalies but exhibits noticeable cross-talk with the density. This is evident from the artifact at the location of the 0.8 density anomaly (green diamond). The multimodal QPAT reduces this cross-talk and provides a more accurate reconstruction of the background, although a slight bias toward the initial guess remains.

**Fig. 7:**
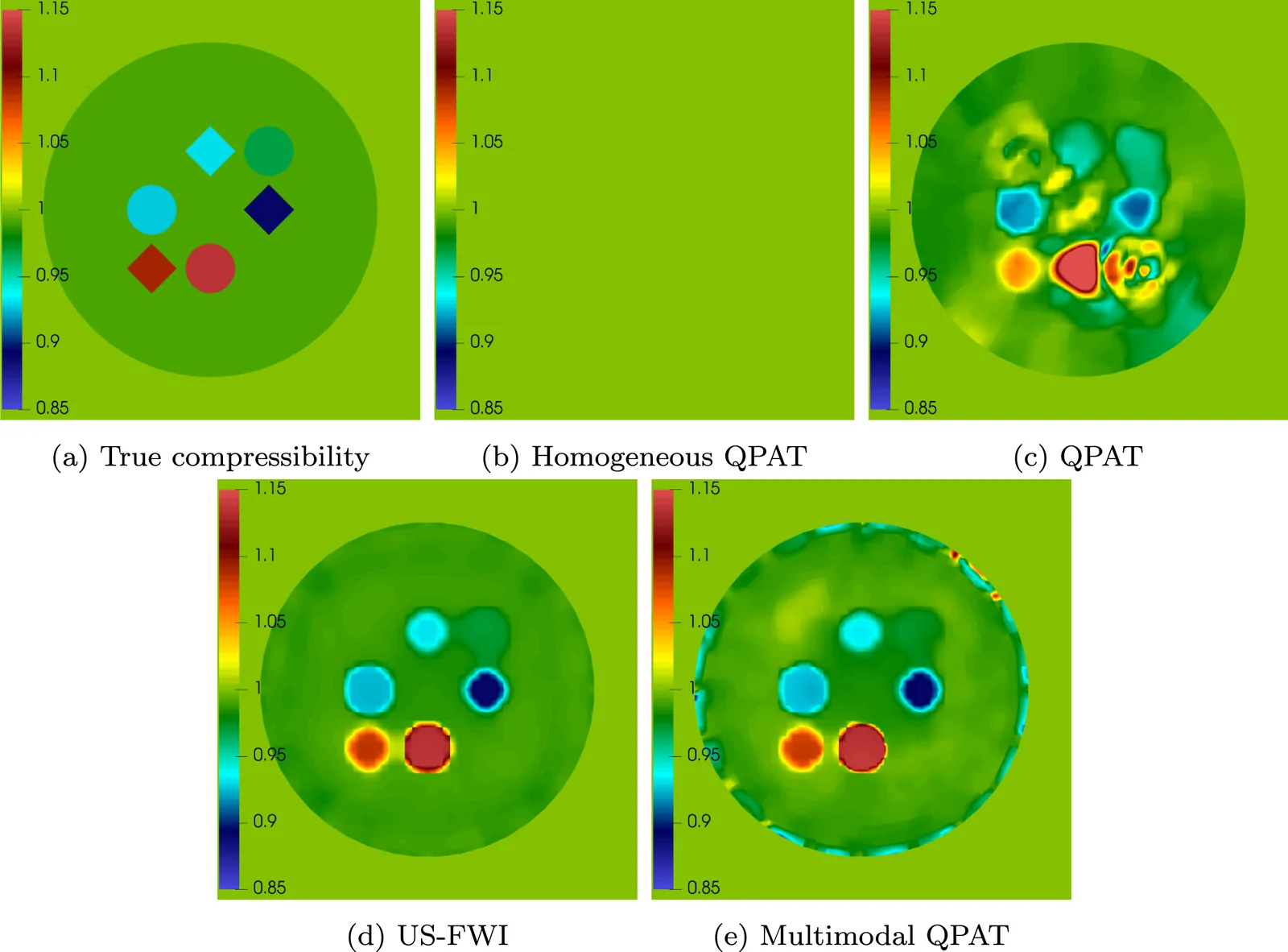
Speed of sound reconstruction results. The speed of sound is shown for completeness and is computed from the inverted compressibility and density rather than being treated as an independent inversion parameter.

For completeness, the reconstructed speed of sound is also shown in Fig. 7. Note that for this particular parameterization, the speed of sound is a derived property from compressibility and density, $c=\sqrt{1/\left(\rho \kappa^{-1}\right)}$. QPAT reconstructs the primary anomalies, although strong cross-talk with the absorption coefficient is evident from the diagonal artifacts at the locations of the absorption anomalies. This suggests that the speed of sound is only weakly identifiable from QPAT measurements, consistent with the findings of [34]. However, the strong cross-talk is likely to make the inversion unstable for more realistic geometries. US-FWI provides the most accurate reconstruction of the speed of sound, as the inversion is not affected by cross-talk with the absorption coefficient. Multimodal QPAT also reconstructs the primary anomalies, although stronger boundary artifacts are observed due to cross-talk with the absorption coefficient.

**Fig. 8:**
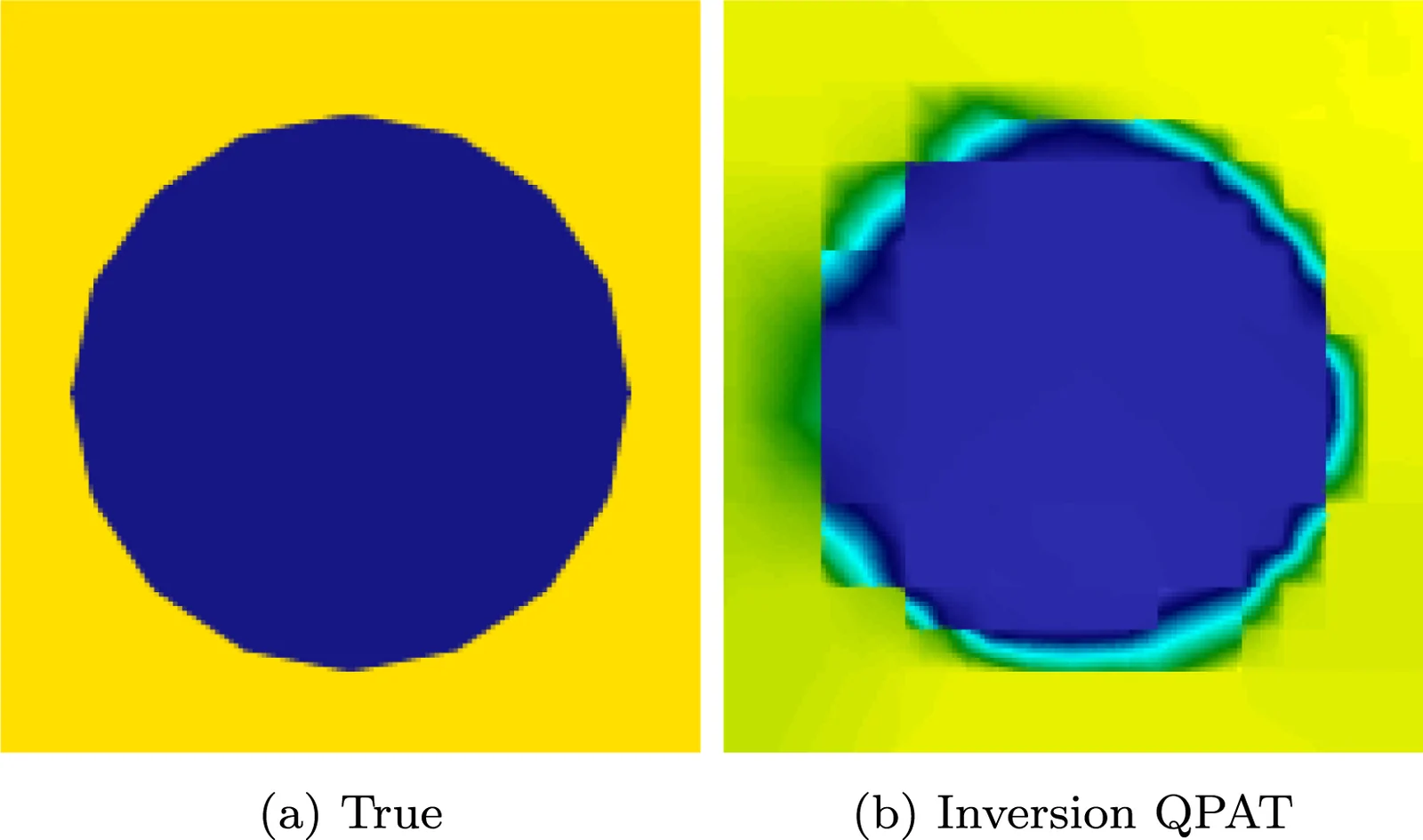
Zoomed-in view of the compressibility inclusion (value 0.8). The reconstruction using linear interpolation polynomials shows inter-cell variation. The result demonstrates how linear interpolation enables subcell resolution.

Increasing the parameterization from piecewise-constant to first-degree polynomials reduces mesh-imposed artifacts by allowing intra-cell variation, as shown in Fig. 8. The inversion mesh was intentionally kept relatively coarse in order to highlight the effect of higher-order parameter representations and to reduce the computational cost of the inversion. Despite the coarse discretization, the use of first-degree polynomial parameter fields enables subcell variation and improves the representation of interfaces that are not aligned with the computational mesh, which is unlikely for material interfaces in biological tissue. Since the parameter fields remain discontinuous between elements, sharp transitions across cell interfaces are still permitted. This demonstrates that the proposed formulation supports higher-order parameter representations while remaining consistent with the Fréchet derivative and inverse Riesz map.

## 7 Discussion

The derivation of the Fréchet derivative based on a general acoustic source term f highlights the versatility of the proposed method. Setting f=0 in (3) recovers conventional QPAT formulations [34], [44], while j=0 in (1) corresponds to time-domain US-FWI [15]. This shows that these approaches can be interpreted as special cases of a single adjoint-state formulation. Although QPAT and US-FWI are typically treated as separate formulations, they arise naturally within the same underlying formulation used here.

As is common in methodological studies of QPAT and waveform inversion, the numerical experiments are intentionally designed to isolate the behaviour of the coupled inversion framework rather than to reproduce a fully realistic imaging scenario. QPAT did not reconstruct the compressibility or density, however the derived property of speed of sound is partially resolved, indicating that speed of sound is weakly identifiably from QPAT measurements with multiple illuminations. However, the inclusion of an acoustic source in multimodal QPAT substantially increased the reconstruction of compressibility and density and therefore speed of sound. The multimodal QPAT shows reduced cross-talk between acoustic parameters compared to US-FWI, suggesting improved separation of the effects of compressibility and density variations. While the inclusion of externally generated wavefields is primarily motivated by improving the conditioning of the optical inversion, the results suggest that photoacoustic sources may also improve the reconstruction of the acoustic parameters, although the mechanism behind this effect is not fully understood.

Material interfaces in biological tissue are unlikely to align with the computational mesh. A piecewise-constant parameterization therefore introduces discretization-induced artifacts, such as staircase boundaries. Allowing higher-degree polynomial variation within elements mitigates this issue by enabling intra-cell gradients, which better approximate arbitrarily oriented interfaces without requiring mesh alignment. Although only first-degree parameter fields are considered in the present numerical examples, the formulation itself is not restricted to a particular polynomial order. Furthermore, higher-order parameter representations are particularly important in waveform inversion problems, where the numerical discretization required for accurate wave propagation does not necessarily coincide with the desired resolution of the inversion parameters. Intra-cell parameter variation therefore improves the representation of material interfaces without requiring uniformly finer computational meshes or substantially increasing the number of inversion parameters.

The implementation contrasts with widely used pseudo-spectral solvers in photoacoustic tomography, such as k-Wave [45], which rely on global spatial FFTs. While efficient on single-node or shared-memory systems, the global nature of FFTs can limit scalability and increase memory demands on distributed-memory architectures.

In three-dimensional settings, memory requirements often become a limiting factor. Global spectral methods require storing the full domain and performing global transforms, whereas the locality of FEM and DG-FEM formulations enables distributed storage and computation, making large-scale simulations more feasible.

Moreover, FEM and DG-FEM naturally support local mesh refinement, allowing computational effort to be concentrated in regions of high sensitivity, which is particularly advantageous in multiscale inversion strategies. Such localized refinement is more difficult to implement efficiently in global spectral methods with uniform resolution.

## 8 Conclusion

A method for inverting acoustic and optical parameters from photoacoustic measurements is presented, based on the adjoint state method. The derivative expressions, obtained via the adjoint method with arbitrary sources, and the optimization procedure are independent of the measurement geometry. The formulation furthermore supports higher-order parameter representations, allowing subcell variation and reducing mesh-alignment artifacts in the reconstructed fields.

Regularization is applied in the form of minimum total variation, motivated by the assumption that tissue consists of regions with nearly constant material properties separated by sharp transitions. An optimization procedure using L-BFGS is employed.

Multiple inversion methods are realized as different source configuration within the same method. These correspond to QPAT, US-FWI, and multimodal QPAT. QPAT is shown to be unstable when reconstructing absorption, density, and compressibility, US-FWI invert the density and compressibility well. However, multimodal QPAT as developed in this work is demonstrated to provide accurate reconstructions of the absorption coefficient, density, and compressibility for a test case using simulated data from a circular array. The speed of sound is obtained as a derived quantity from the reconstructed density and compressibility. This indicates potential for future studies on tissue functionalization.

No significant degradation was observed for the considered level of additive Gaussian noise. This is consistent with the least-squares formulation employed in the inversion. However, the robustness of the method to higher noise levels was not assessed.

## CRediT authorship contribution statement

**Manne Segerlund:** Writing – original draft, Visualization, Software, Methodology, Formal analysis. **Torbjörn Löfqvist:** Writing – review & editing, Supervision, Conceptualization.

## Declaration of competing interest

The authors declare that they have no known competing financial interests or personal relationships that could have appeared to influence the work reported in this paper.

## Data Availability

No data was used for the research described in the article.

## Appendix A Operator-level linearization and adjoint derivations

The objective function defined in Section 3 can be rewritten as an inner product,

$$J=\frac{1}{2}\|P\left(u\right)-d{\|}^{2}={\langle 1,\;\chi \left(u\right)\rangle }_{D}.$$

Here, χ denotes a (generally nonlinear) operator acting on the state u, and 1 denotes a constant unit function on the data space. The subscript D denotes the inner product in the data space. This formulation admits any objective functional that can be expressed through a data space misfit density χ. Modifying the objective functional therefore changes only the adjoint source terms appearing in the adjoint acoustic equation.

The notation D is used to denote Fréchet derivative of an operator, to distinguish it from the gradient of a function, which is denoted by ∇. As in [46] the derivative of the objective function begins by identifying,

$$D_{m}J\;\delta m={\langle 1,\;D_{m}\chi \left(u\right)\;\delta m\rangle }_{D}={\langle 1,\;D_{u}\chi \left(u\right)\left[D_{m}u\;\delta m\right]\rangle }_{D}={\langle D_{u}\chi {\left(u\right)}^{*}\left[1\right],\;\delta u\rangle }_{S}$$

by invoking the chain rule, where

$$\delta u\overset{\text{def}}{=}D_{m}u\;\delta m.$$

With subscript S for inner product of the acoustic states. Following the continuation in [46], this yields

(A.1) $$D_{m}J\left(m\right)\;\delta m={\langle u^{*},\;D_{m}L_{A}\left(u,m\right)\;\delta m\rangle }_{S},$$

where $u^{*}=\left(p^{*},v^{*}\right)$ denotes the solution of the adjoint problem

(A.2) $$D_{u}L_{A}{\left(u,m\right)}^{*}\;u^{*}=-D_{u}\chi {\left(u\right)}^{*}\left[1\right].$$

Next, the derivative with respect to the model parameters associated with the optical inversion is considered. Let $m_{\phi }$ represent the model parameters associated with the optical inversion problem, $m_{\phi }=\left(\mu ,D,\Gamma \right)\in {\left(L^{2}\left(\Omega \right)\right)}^{3}$. Here, the absorption coefficient, diffusion coefficient, and Grüneisen parameter. Then, let

$$p_{0}^{*}\left(x\right)\overset{\text{def}}{=}p^{*}\left(x,t_{0}\right)$$

be the solution of the adjoint wave equation at $t_{0}$, which will be the final time-step of the simulation [47]. The photoacoustic initial pressure may be interpreted as a source term acting at $t=t_{0}$,

$$s\left(x,t\right)=\delta \left(t-t_{0}\right)p_{0}\left(x\right).$$

The derivative of the objective functional with respect to the optical parameters may therefore be expressed through the derivative with respect to this source term. Following [46],

(A.3) $$D_{m_{\phi }}J\;\delta m_{{}_{\phi }}=D_{p_{0}}J\;\delta p_{0}=-\langle p_{0}^{*},\;\delta p_{0}\rangle .$$

where the directional derivative $\delta p_{0}$ with respect to $m_{\phi }$ is

$$\delta p_{0}\overset{\text{def}}{=}D_{m_{\phi }}p_{0}\;\delta m_{\phi }=\phi D_{m_{\phi }}\left(\Gamma \mu \right)\;\delta m_{\phi }+\Gamma \mu D_{m_{\phi }}\left(\phi \right)\;\delta m_{\phi }.$$

Note that the definition of $\delta p_{0}$ implicitly assumes stress confinement. For convenience, we define

$$\delta \phi \overset{\text{def}}{=}D_{m_{\phi }}\phi \;\delta m_{\phi },$$

viewing $\phi \left[m_{\phi }\right]$ as an operator mapping material parameters to the solution of the photon diffusion Partial Differential Equation (PDE). Inserting this into (A.3) gives

(A.4) $$D_{m_{\phi }}J\delta m_{{}_{\phi }}=-\langle p_{0}^{*},\;\phi D_{m_{\phi }}\left(\Gamma \mu \right)\;\delta m_{\phi }\rangle -\langle p_{0}^{*},\;\Gamma \mu \;\delta \phi \rangle .$$

The first part is easy to calculate for any $m_{\phi }$, as the derivative of μ with respect to μ is the identity operator and the same holds for Γ. For the second part, recall the photon diffusion equation satisfies

$$L_{\phi }\left(\phi ,m_{\phi }\right)=0.$$

Differentiating this expression with respect to $m_{\phi }$ and invoking the chain rule yields

$$D_{m_{\phi }}L_{\phi }\;\delta m_{\phi }+D_{\phi }L_{\phi }\;\delta \phi =0.$$

Multiply with an arbitrary test function $\phi^{*}$ and integrate,

$$\langle \phi^{*},\;D_{m_{\phi }}L_{\phi }\delta m_{\phi }\rangle +\langle \phi^{*},\;D_{\phi }L_{\phi }\delta \phi \rangle =0.$$

Assuming that an adjoint operator to $D_{\phi }L$ exist and rearranging,

$$\langle \phi^{*},\;D_{m_{\phi }}L_{\phi }\delta m_{\phi }\rangle +\langle D_{\phi }L_{\phi }^{*}\phi^{*},\;\delta \phi \rangle =0$$

and adding to (A.4) gives

(A.5) $$D_{m_{\phi }}J\delta m_{{}_{\phi }}=-\langle p_{0}^{*},\;\phi D_{m_{\phi }}\left(\Gamma \mu \right)\;\delta m_{\phi }\rangle -\langle \Gamma \mu p_{0}^{*}-D_{\phi }L_{\phi }^{*}\phi^{*},\;\delta \phi \rangle +\langle \phi^{*},\;D_{m_{\phi }}L_{\phi }\delta m_{\phi }\rangle .$$

If $\phi^{*}$ fulfils the equation

$$D_{\phi }L_{\phi }^{*}\phi^{*}=\Gamma \mu p_{0}^{*}$$

(A.5) reduces to

$$D_{m_{\phi }}J\delta m_{{}_{\phi }}=-\langle p_{0}^{*},\;\phi D_{m_{\phi }}\left(\Gamma \mu \right)\delta m_{\phi }\rangle +\langle \phi^{*},\;D_{m_{\phi }}L_{\phi }\delta m_{\phi }\rangle .$$

Since $L_{\phi }$ is linear in ϕ it follows that $L_{\phi }^{*}$ is also linear in ϕ and the adjoint equation reads

(A.6) $$L_{\phi }^{*}\phi^{*}=\Gamma \mu p_{0}^{*}$$

which gives the expression for the derivative of J with respect to optical model parameters. Combining it all together, the derivative with respect to a model parameter m can then be calculated as

(A.7) $$D_{m}J\;\delta m=\langle u^{*},\;D_{m}L_{A}\;\delta m\rangle -\langle p_{0}^{*},\;\phi \;D_{m}\left(\Gamma \mu \right)\;\delta m\rangle +\langle \phi^{*},\;D_{m}L_{\phi }\;\delta m\rangle$$

given u and $p_{0}^{*}$ solves the acoustic adjoint equation, and $\phi^{*}$ solves the photon diffusion adjoint equation. Here, no assumption about the specific optical or acoustic governing equation is made; $L_{A}$ and $L_{\phi }$ can represent any linear PDE for which a well-defined adjoint operator exists, and the derivation remains valid. Note that the only model-specific assumption is stress-confinement, $p_{0}=\Gamma \mu \phi$, linking the optical and acoustic problems.

From here on, the focus will be on the adjoint equations for the specific models chosen in this paper. The derivation for the acoustic adjoint state method is found elsewhere, e.g. [48], and only the resulting equations will be presented here. The adjoint equation becomes

$$\left\{\begin{array}{c} \alpha \frac{\partial p^{*}}{\partial t}+\nabla \cdot v^{*}=q_{p}\;\\ \beta \frac{\partial v^{*}}{\partial t}+\nabla p^{*}=q_{v}\;\\ p^{*}\left(x,T_{f}\right)=0\;\\ v^{*}\left(x,T_{f}\right)=0\; \end{array}\right)$$

with

$$\left(q_{p},q_{v}\right)=P^{*}\left(P\left(u\right)-d\right)$$

where P is the observer operator that maps the pressure field to the data domain and $P^{*}$ is the adjoint operator to P for the least-squares objective function. The adjoint equation is the regular acoustic equation with a final value. This final value equation can be rewritten using a change of variable of $t^{\prime }\mapsto T_{f}+t_{0}-t$ to an initial value problem and the adjoint state is then formulated as

$$\left(\begin{array}{c} {\overset{ˆ}{p}}^{*}\left(x,t^{\prime }\right)\\ {\overset{ˆ}{v}}^{*}\left(x,t^{\prime }\right) \end{array}\right)=\left(\begin{array}{c} p^{*}\left(x,T_{f}+t_{0}-t^{\prime }\right)\\ -v^{*}\left(x,T_{f}+t_{0}-t^{\prime }\right) \end{array}\right)$$

giving the new adjoint equation as

(A.8) $$\left\{\begin{array}{c} \alpha \frac{\partial {\overset{ˆ}{p}}^{*}}{\partial t^{\prime }}+\nabla \cdot {\overset{ˆ}{v}}^{*}=-q_{p}\left(T_{f}+t_{0}-t^{\prime }\right)\;\\ \beta \frac{\partial {\overset{ˆ}{v}}^{*}}{\partial t^{\prime }}+\nabla {\overset{ˆ}{p}}^{*}=q_{v}\left(T_{f}+t_{0}-t^{\prime }\right)\;\\ {\overset{ˆ}{p}}^{*}\left(x,0\right)=0\;\\ {\overset{ˆ}{v}}^{*}\left(x,0\right)=0\; \end{array}\right)$$

which is the acoustic wave equation running backwards in time from $T_{f}$ to $t_{0}$ with the residual P(u)−d injected as a source term using $P^{*}$.

Let δϕ denote a perturbation to the photon fluence ϕ. Since ϕ satisfies the Robin boundary condition ϕ+2D∇ϕ⋅n=j, the perturbation must satisfy the homogeneous Robin condition,

δϕ+2D∇(δϕ)⋅n=0,on∂Ω,

such that ϕ+δϕ continues to satisfy the original boundary condition.

Using integration by parts twice, the adjoint equation can be derived as,

$$\langle L_{\phi }\delta \phi ,\;\phi^{*}\rangle =\int_{\Omega }\left(\mu \delta \phi -\nabla \cdot \left(D\nabla \delta \phi \right)\right)\phi^{*}dV=\int_{\Omega }\delta \phi \left(\mu \phi^{*}-\nabla \cdot \left(D\nabla \phi^{*}\right)\right)dV-\int_{\partial \Omega }D\nabla \delta \phi \cdot n\phi^{*}-D\delta \phi \;\nabla \phi^{*}\cdot ndA=\langle \delta \phi ,\;L_{\phi }^{*}\phi^{*}\rangle .$$

Which gives the adjoint equation. For the Robin boundary condition,

$$\int_{\partial \Omega_{R}}D\nabla \delta \phi \cdot n\phi^{*}-D\delta \phi \;\nabla \phi^{*}\cdot ndA=\int_{\partial \Omega_{R}}-\frac{1}{2}\delta \phi \phi^{*}-D\delta \phi \;\nabla \phi^{*}\cdot n\;dA=\int_{\partial \Omega_{R}}-\delta \phi \left(\frac{1}{2}\phi^{*}+D\nabla \phi^{*}\cdot n\right)dA=0,\forall \;\delta \phi .$$

If the adjoint radiative field $\phi^{*}$ fulfils the homogeneous Robin boundary condition the boundary term will cancel out for all perturbations δϕ. The adjoint state equation with boundary conditions reads

(A.9) $$\left\{\begin{array}{cc} \mu \phi^{*}-\nabla \cdot \left(D\nabla \phi^{*}\right)=\Gamma \mu p_{0}^{*}\; & \text{in}\;\Omega \\ \phi^{*}+2D\nabla \phi^{*}\cdot n=0\; & \text{on}\;\partial \Omega . \end{array}\right)$$

## Appendix B Measurement operator and adjoint

The observer operator and its adjoint is defined as:

$$P\;:\;p\mapsto \left\{p_{ij}\right\},\;p_{ij}=\int_{t_{0}}^{T_{f}}\int_{\Omega }\overset{ˆ}{\delta }\left(x-x_{j}\right)\frac{1}{\Delta t}\theta_{i}\left(t\right)p\left(x,t\right)dVdt$$

$$P^{*}\;:\;\left\{a_{ij}\right\}\mapsto q_{p},\;q_{p}\left(x,t\right)=\overset{N_{t}}{\underset{i=1}{\sum }}\overset{N_{s}}{\underset{j=1}{\sum }}\overset{ˆ}{\delta }\left(x-x_{j}\right)\theta_{i}\left(t\right)a_{ij}.$$

 Here, $\overset{ˆ}{\delta }$ denotes a smooth approximation of point evaluation at the sensor location $x_{j}$, and $\theta_{i}\left(t\right)$ is a square pulse supported on the interval $\left[t_{i},t_{i+1}\right)$ of width Δt.

We equip the data space with the discrete inner product

$${\langle a,\;b\rangle }_{D}=\overset{N_{t}}{\underset{i=1}{\sum }}\overset{N_{s}}{\underset{j=1}{\sum }}a_{ij}b_{ij}\;\Delta t,$$

The objective function takes the form of,

$$J=\Delta t\overset{N_{t}}{\underset{i=1}{\sum }}\overset{N_{s}}{\underset{j=1}{\sum }}{\left(H_{f}{\left(P\left(u\right)-d\right)}_{ij}\right)}^{2}$$

and the adjoint source is given by

$$\left(q_{p},q_{v}\right)=P^{*}\left(H_{f}\left(P\left(u\right)-d\right)\right).$$

The low pass filter is defined as a Gaussian taper in the frequency domain as,

$$H_{f}\left(f\right)=\left\{\begin{array}{cc} 1\; & \text{if}\;\left|f\right|<f_{c}\\ \exp\left(-\frac{1}{2}{\left(a\frac{\left|f\right|-f_{c}}{0.5\Delta f}\right)}^{2}\right)\; & \text{if}\;\left|f\right|\geq f_{c}, \end{array}\right)$$

where $f_{c}$ is the cutoff frequency and Δf controls the taper width.
